# Sodium and Potassium Intake from Food Diaries and 24-h Urine Collections from 7 Days in a Sample of Healthy Greek Adults

**DOI:** 10.3389/fnut.2018.00013

**Published:** 2018-02-21

**Authors:** Adelais Athanasatou, Aikaterini Kandyliari, Olga Malisova, Alex Pepa, Maria Kapsokefalou

**Affiliations:** ^1^Unit of Human Nutrition, Department of Food Science and Human Nutrition, Agricultural University of Athens, Athens, Greece

**Keywords:** sodium intake, potassium intake, 24-h urine excretion, food diaries, 6-h interval samples

## Abstract

**Objective:**

The main objective of the present study was to evaluate sodium and potassium intake, employing 24 h and spot urine samples and food diaries for seven consecutive days.

**Methods:**

For seven consecutive days subjects recorded their food and drink intakes, and recorded and collected all urinations. Food sodium and potassium intake were analyzed in 24- and 6-h intervals from wake-up time. Urine indices were analyzed in first morning, 24- and 6-h intervals samples over the day from wake-up time. The study took place in Agricultural University of Athens, Greece. In total, 163 healthy subjects (age 39 ± 12 years; 74 females) were enrolled in the study.

**Results:**

Mean urine sodium excretion was 2,803.3 ± 1,249.0 mg/day (121.9 ± 54.3 mmol/day) and mean urine potassium excretion was 2,152.2 ± 913.3 mg/day (55.2 ± 23.4 mmol/day). The highest potassium concentration was measured in the afternoon, while the lowest sodium concentration was measured in the overnight 6-h interval. Food sodium intake was 1,983.2 ± 814.1 mg/day and food potassium was 2,264.5 ± 653.3 mg/day. The sources that contribute most in food sodium intake are dairy products 24%, breads 22%, and savory snacks 17%.

**Conclusion:**

Strategies should encourage the Greek population to moderate sodium intake and promote potassium intake, thus adopting a healthier dietary and lifestyle pattern.

## Introduction

Strong and consistent evidence suggests that high-sodium and low-potassium dietary intakes are associated with increased blood pressure (1–4). Hypertension is a major risk factor for strokes, coronary, and cardiovascular diseases (5–7), resulting in 9.4 million deaths annually (8). Yet, excessive sodium consumption remains a major public health problem worldwide (9). In response to available evidence, advice on reduction of salt intake has been issued. The Dietary Approach to Stop Hypertension (DASH) diet recommends a lower intake of sodium, sugars and fats, and a higher intake of potassium, calcium, and magnesium (10). The World Health Organization (WHO) suggests that adults should consume less than 2,000 mg of sodium, or 5 g of salt, and at least 3,510 mg of potassium per day (11). The Center for Disease Control and Prevention (CDC) indicates that 90% of children and 89% of adults consume more sodium than it is suggested (12).

The main source of sodium in our diet is salt. Salt is widely consumed as a common ingredient in most processed foods and as an additive during cooking and at the table (13, 14). Depending on the dietary habits of the population, the sources of salt intake vary. For example, in the US bakery products, meat/cold cuts, and pizza, while in Asian countries seasoning such as soy sauce, and miso, contribute more to the total salt consumption (15, 16). The main sources of potassium intake are fruits and vegetables (17), but a decreased consumption has been recorded (18).

Sodium and potassium intakes are evaluated from dietary assessment methodologies, particularly food-frequency questionnaires and food diaries. These have been employed in studies conducted in many countries (19) including Greece (20, 21); however, these methods may underestimate salt intake (19). Intake of sodium and potassium is measured more accurately in urine samples collected over 24 h (22). This approach has been used, for example, in the UK in order to evaluate the strategy toward sodium reduction (23), in the INTERSALT study in order to measure sodium intake in 29 North American and European populations (24) and in Northern Greece in order to evaluate sodium intake in the population (25). Some studies, to overcome the burden of 24-h urine collection, use spot urine samples, such as first morning samples, to extrapolate to the 24-h sodium and potassium urine excretion (24, 26–28). These procedures may mask fluctuations in sodium and potassium intake that occur during the day or in periods longer than a day. Observation on sodium and potassium intake from urine samples collected during the day and in periods longer than a day, such as in seven consecutive days, are necessary to further understand patterns on sodium and potassium intake, correlate with dietary habits and support improvement in methodologies employed in the evaluation of sodium and potassium intake.

The objectives of the present study were (a) to measure sodium and potassium in 24-h urine samples for seven consecutive days, (b) to measure sodium and potassium in spot urine samples (first morning, 6-h intervals) for three consecutive days, (c) to estimate sodium and potassium intake from foods using 7-day food diaries, and (d) to evaluate the contribution of foods groups to sodium intake in a sample of healthy Greek adults.

## Materials and Methods

In the framework of the European Hydration Research Study (EHRS) (29), we studied a subsample of 163 Greek subjects from the metropolitan area of Athens enrolled to the study during winter (12/2013, 1-2/2014) and summer (7-8/2013, 6-7/2014). Subjects were adults aged 20–60 years with approximately equal numbers in each decade of life. Exclusion criteria were disease (diabetes insipidus, renal disease, liver disease, gastrointestinal diseases or problems, cardiac or pulmonary diseases, disease that limits mobility including muscle-skeletal diseases, or orthopedic problems), pregnancy, lactation, hypertensive under severe salt restriction, taking drugs that are, or contain, diuretics, phenytoin, lithium, demeclocycline, or amphotericin, and following a high-protein and/or hypocaloric diet. Subjects were rescheduled or omitted if they caught flu (cold) or had fever, vomiting, and/or diarrhea or menstruation during the data collection period. Urinary volumes <500 mL and creatinine excretion rate (CER) >3,500 mg/day or <350 mg/day suggest inaccurate urine collection (30). Nine subjects were excluded from the studied population for non-compliance to the protocol. Energy intake was not considered as an exclusion criterion for the study. The recruitment strategy included invitations (a) sent by email to the non-academic and academic personnel, (b) uploaded on social media and published in local newspapers, (c) uploaded on internet sites related to nutrition, (d) distributed in paper at various non-academic places, (e) sent by email to other academic and social work institutions in the greater area, and (f) distributed at any seminar that the research teams were giving. The response rate was approximately 10%. The recruitment strategy performed in a random sample of the population and the subjects that responded to the invitations were categorized according to their age and sex.

This study was carried out in accordance with the recommendations of the Research Ethics Committee of Agricultural University of Athens, Greece (197/27-02-2012). This study was approved by the Research Ethics Committee of Agricultural University of Athens, Greece. All subjects gave written informed consent in accordance with the Declaration of Helsinki (31). Details that might disclose the identity of the subjects under study were omitted.

### Experimental Design

This observational study (evaluating sodium and potassium intake) was carried out for seven consecutive days. We decided to divide the 24-h period of the last 3 days of the study in 6-h intervals: morning (0–6 h from wake-up time); afternoon (6–12 h from wake-up time); evening (12–18 h from wake-up time); and overnight (18–24 h from wake-up time) in order to measure in details, the sodium and potassium intake and excretion throughout the day.

Subjects were instructed to collect and record the weight of each urination and the time of collection and to retain each sample in a numbered tube. Subjects stored the urine tubes in styrofoam box using ice packs until arrival to the refrigerator. A 24-h-reconstituted sample of 10 mL was prepared in the laboratory by pooling these urine samples in a volume proportional addition of each specimen for each day of the study. A 10-mL-reconstituted sample was also prepared for each 6-h interval, consisted of all samples that were collected during the 6-h period by volume proportional addition of each specimen for the last 3 days of the study. In addition, first morning urine (FMU) samples were collected separately. Moreover, subjects recorded the type and amount of food and/or fluid consumption, time and place, immediately after it happened, in order to avoid misreporting. Subjects were instructed by a dietician, *via* personal interview, before the initiation of the study to estimate the portion sizes and report it in their food diaries; pictures of portion sizes for foods groups usually consumed in Greece were included in food diaries. Subjects were also counseled to maintain their usual physical activity, eating, and drinking habits.

Analyses in urine were carried out on first morning samples, 24-h collection and 6-h interval samples. Urine sodium and potassium were measured by ion selective electrode methods (Cobas Integra 400 plus). Indices for accuracy of the 24-h urine collection were urine volume, measured with an electronic digital scale (Soehnle Fiesta 65106) and urine creatinine, measured by the Jaffé enzymatic colorimetric method (Cobas Integra 400 plus). Sodium and potassium intakes from 7-day diaries were analyzed with Diet Analysis plus version 6.1software (ESHA Research, Wadsworth Publishing Co Inc., Salem, OR, USA); thus, the contribution of table salt to total sodium intake was not estimated.

### Statistical Analysis

Continuous variables are expressed as mean ± SD for variables following normal distribution and as median (P25, P75) for the skewed variables. Normal distribution of all continuous variables was tested with the parametric test Shapiro–Wilk or graphically assessed by histograms. Correlations between variables were evaluated using Pearson’s or Spearman’s correlation coefficient. Differences between genders *P* were derived through Student’s *t*-test for normally distributed variables and Mann–Whitney *U* test for the skewed variables. Differences among 6-h intervals were derived through one-way ANOVA test for normally distributed variables. *Post hoc* comparisons among 6-h intervals were performed using Bonferroni’s test. The multivariate associations between variables were assessed using linear regression models, adjusted for all biologically plausible confounders.

The measured 24-h urine indices were calculated as follows:

$$\begin{array}{l} 24\text{-h urinary sodium excretion }(\text{mg/day})=\text{concentration of}\\ \;24\text{-h urinary sodium excretion }(\text{mmol/L})\\ \;\times \;24\text{-h urine volume }(\text{L/day})\\ \;\times \text{ molecular weight of}\;{\text{Na}}^{+}(\text{23 mg/mmol}) \end{array}$$

$$\begin{array}{l} 24\text{-h urinary potassium excretion }(\text{mg/day})=\text{concentration of}\\ \;24\text{-h urinary potassium excretion }(\text{mmol/L})\\ \;\times 24\text{-h urine volume }(\text{L/day})\\ \;\times {\text{ molecular weight of K}}^{+}(\text{39 mg/mmol}) \end{array}$$

Statistical analysis was performed by SPSS package, version 16.1 (SPSS Inc., Chicago, IL, USA). We deemed statistical significance at α = 0.05.

## Results

The studied population consisted of 163 subjects (age 39 ± 12 years; 74 females). The mean BMI of males was 25.6 ± 4.8 kg/m^2^ and females 24.5 ± 4.5 kg/m^2^ (*p* = 0.149).

In Table 1, mean data from 7-day diaries (total energy, food sodium, and food potassium intake) and 24-h urine samples from seven consecutive days (volume, sodium, potassium, and creatinine) for males, females, and totally are presented. Males had higher energy and food sodium intake than females (*p* < 0.05 in both cases). Food sodium intake was positively correlated with energy intake (*r* = 0.702, *p* < 0.001) and food potassium intake (*r* = 0.390, *p* < 0.001). Food potassium was also correlated positively with energy intake (*r* = 0.427, *p* < 0.001). During the 7 days, 24-h urine sodium correlated with 24-h urine potassium (*r* = 0.657, *p* < 0.001). The 24-h urine sodium correlated with food sodium intake (*r* = 0.334, *p* < 0.001), and total energy intake (*r* = 0.363, *p* < 0.01). The 24-h urine potassium correlated with energy intake (*r* = 0.242, *p* = 0.002) and food potassium (*r* = 0.297, *p* < 0.001). No differences were observed in food potassium intake, urine volume, sodium, potassium, and creatinine urine concentration between males and females and between summer and winter periods.

**Table 1:** Data from 7-day diaries and 24-h urine collection samples of subjects for seven consecutive days.

	Total (*n* = 163)		Male (*n* = 89)		Female (*n* = 74)		*P*
	Mean	SD	Mean	SD	Mean	SD	
Energy intake (kcal/day)	1,732	511	1,807	560	1,642	433	0.037
Food sodium intake (mg/day)	1,983	814	2,102	897	1,841	680	0.036
Food potassium intake (mg/day)	2,265	653	2,345	667	2,167	627	0.084
Urine volume (L/day)	1.25	0.50	1.23	0.48	1.28	0.53	0.557
Urine Na (mg/day)	2,803.3	1,249.0	2,872.7	1,366.4	2,721.3	1,098.1	0.454
Urine K (mg/day)	2,152.2	913.3	2,084.9	910.6	2,231.8	916.5	0.320
Urine creatinine (mg/day)	121.6	48.0	125.3	51.0	117.2	44.1	0.289

*Results are presented as mean (SD). *P*-values derived through Student’s *t*-test for differences between genders*.

In Table 2, urine indices (volume, sodium, potassium, and creatinine excretion) for 6-h intervals (morning, afternoon, evening, and overnight samples), FMU, and 24-h urine samples for three consecutive days, for males, females, and totally are presented. Urine sodium excretion in the overnight period was higher in males (75.0 ± 208.4; 2.8 ± 6.8 mg/time interval) than in females (31.8 ± 86.8; 1.2 ± 3.2 mg/time interval) (*p* < 0.05 in both cases). Differences were observed among 6-h intervals for all urine indices [volume *F*(3, 636) = 126.983 *p* < 0.001; sodium *F*(3, 521) = 19.716 *p* < 0.001; potassium *F*(3, 521) = 42.570 *p* < 0.001; creatinine *F*(3, 521) = 14.914 *p* < 0.001]. *Post hoc* tests using the Bonferroni’s correlation revealed that urine potassium concentration in the afternoon interval was higher as compared with the other 6-h intervals. Males excreted higher amounts of sodium compared with women in the afternoon interval. Urine sodium in the morning interval reflects by 34% of urine sodium in the 24-h sample, while urine potassium in the evening interval reflects by 31% the 24-h sample.

**Table 2:** Volume, sodium, potassium, and creatinine excretion in four 6-h intervals starting at wake-up time, 24 h, and first morning urine (FMU) samples.

	Morning (0–6 h)		Afternoon (6–12 h)		Evening (12–18 h)		Overnight (18–24 h)		24 h		FMU	
						
	Mean	SD	Mean	SD	Mean	SD	Mean	SD	Mean	SD	Mean	SD
**Total volume (L)**												
Total	0.53	0.28	0.36	0.22	0.27	0.17	0.08	0.14	1.24	0.56	0.33	0.12
Male	0.52	0.27	0.36	0.23	0.27	0.18	0.08	0.13	1.21	0.54	0.33	0.13
Female	0.53	0.29	0.36	0.22	0.26	0.16	0.08	0.14	1.28	0.59	0.33	0.11

**Urine Na (mg/time interval)**												
Total	1,018.8	690.1	821.7	704.4	551.5	439.7	55.9	166.8	2,902.9	1,344.0	693.4	507.3
Male	1,074.9	727.6	920.9	829.5	565.0	486.6	75.0*	208.4	3,028.0	1,480.4	671.4	340.6
Female	949.0	638.8	701.1*	493.0	534.0	373.4	31.8	86.8	2,759.1	1,162.3	719.1	652.2

**Urine K (mg/time interval)**												
Total	727.2	520.2	713.5	524.5	389.2	319.1	38.1	120.5	2,215.2	1,064.1	443.4	250.6
Male	744.4	518.2	735.6	564.7	389.9	349.4	49.0	147.7	2,167.3	1,059.9	446.6	247.3
Female	705.8	525.6	686.7	473.8	388.2	277.7	24.3	72.2	2,270.9	1,074.2	439.5	256.8

**Urine creatinine (mg/time interval)**												
Total	49.4	48.8	34.1	33.9	24.4	23.5	2.1	5.5	121.4	52.4	40.6	51.2
Male	49.1	41.6	34.0	33.2	24.2	25.6	2.8*	6.8	123.3	57.4	35.1	17.0
Female	49.6	56.8	34.2	35.0	24.7	20.5	1.2	3.2	119.2	46.1	47.7	74.9

*Results are presented as mean (SD). *P*-values derived through Student’s *t*-test for differences between genders and one-way ANOVA among 6-h intervals*.

*p < 0.05 indicates significant difference between males and females.

In Table 3, data from the 3-day dairies (total energy, food sodium, and food potassium intake) for 6-h intervals (morning, afternoon, evening, and overnight samples), and 24 h for males, females, and totally are presented. Mean energy intake was 1,686 ± 610 kcal/day, food sodium intake was 2,064.26 ± 1,006.78 mg/day, and food potassium intake was 2,366.91 ± 793.10 mg/day. Food sodium intake correlated with age (*r* = −0.339, *p* < 0.001) and with food potassium intake (*r* = 0.318, *p* < 0.001). Sodium intake density (mg Na/1,000 kcal), using data from Table 3, allows comparisons of sodium intake without cofounding related to total caloric intake. Food sodium density in 24-h periods for the total sample, males, and females was 1,430, 1,239, and 1,671 mg/1,000 kcal, respectively. Also, potassium intake density (mg K/1,000 kcal) was 1,872, 1,454, and 2,399 mg/1,000 kcal for the total sample, male, and females, respectively.

**Table 3:** Energy, food sodium, and food potassium intake of subjects in three consecutive days in four 6-h intervals starting at wake-up time and over a 24-h period.

	Morning (0–6 h)		Afternoon (6–12 h)		Evening (12–18 h)		Overnight (18–24 h)		24 h	
					
	Mean	SD	Mean	SD	Mean	SD	Mean	SD	Mean	SD
**Energy intake (kcal/time interval)**										
Total	652	356	692	314	511	292	181	186	1,686	610
Male	674	379	699	300	553	311	207	218	1,792*	671
Female	627	327	684	332	459	260	126	72	1,561	507

**Food sodium intake (mg/time interval)**										
Total	757	535.58	776.18	515.39	517.48	389.04	89.22	126.04	2,064.26	1,006.78
Male	783.1	577.5	766.3	569.9	558.0	425.0	108.1	142.9	2,124.80	1,172.05
Female	724.32	479.58	788.65	335.14	466.88	335.14	53.41	79.78	1,987.69	749.15

**Food potassium intake (mg/time interval)**										
Total	877.47	492.41	913.52	460.67	581.39	355.58	139.25	147.02	2,366.91	793.10
Male	882.0	513.9	898.6	516.1	582.0	333.3	174.3	145.9	2,362.6	874.2
Female	871.73	467.52	932.35	382.13	580.60	384.84	100.26	146.41	2,372.33	683.20

Results are presented as mean (SD). *P*-values derived through Student’s *t*-test for differences between genders and one-way ANOVA among 6-h intervals. * *p* < 0.05 indicates significant difference between males and females.

Sodium intake from 3-day food diaries correlated with sodium measured in 24 h (*r* = 0.209, *p* < 0.05) in morning (*r* = 0.168, *p* < 0.05), and in evening (*r* = 0.212, *p* < 0.05) urine. Furthermore, sodium intake in the evening interval correlated with sodium in evening urine sample (*r* = 0.188, *p* < 0.05). Potassium intake from 3-day diaries correlated with potassium measured in 24 h (*r* = 0.266, *p* < 0.01), in morning (*r* = 0.192, *p* < 0.05), in afternoon (*r* = 0.263, *p* < 0.01), and in evening (*r* = 0.208, *p* < 0.05) urine.

Differences were observed among 6-h intervals for energy intake [*F*(3, 503) = 24.711 *p* < 0.001], food sodium intake (*F*(3, 486) = 24.044 *p* < 0.001), and food potassium intake [*F*(3, 471) = 31.512 *p* < 0.001]. *Post hoc* tests using the Bonferroni’s correlation revealed that energy intake in the evening and in the overnight (511 ± 292 and 181 ± 186 kcal, respectively) interval was significantly lower (*p* < 0.001) than the in the morning and in the afternoon interval (652 ± 356 and 692 ± 314 kcal, respectively). No differences were observed in food sodium and potassium intake among males and females in 6-h intervals (*p* > 0.005 in all time intervals).

The intake of sodium and potassium in the morning (757.16 ± 535.58 and 877.47 ± 492.41 mg, respectively) and in the afternoon intervals (776.18 ± 515.39 and 913.52 ± 460.67 mg, respectively) was significantly higher (*p* < 0.001) than in the evening (517.48 ± 389.04 and 581.39 ± 355.58 mg, respectively) and in the overnight interval (89.22 ± 126.04 and 139.25 ± 147.02 mg, respectively).

Mean daily food sodium intake from 7-day diaries was 1,983.2 ± 814.1 mg/day and food potassium was 2,264.5 ± 653.3 mg/day. The sources of food sodium intakes that contributed higher than 3% of the total food sodium intakes are presented in Table 4. The main contributors to sodium intake were the dairy products (including cheese, yogurt, and milk), breads (including cereals, crackers, rusks, and toasted bread) and savory snacks. In descending order, the contributors to food sodium intake were poultry, pizza, cold cuts, pasta mixed dishes, sandwiches, meat mixed dishes, oils, soups, fish, eggs, and beverages. Significant differences were observed in food sodium intake from savory snacks (*p* = 0.013), pasta mixed dishes (*p* = 0.031), meat mixed dishes (*p* = 0.006), and fish (*p* = 0.046) between males and females.

**Table 4:** Sources of food sodium intake from 7-day diaries of males and females.

	Food sodium intake (mg) per food group			
	Total (*n* = 163)	Male (*n* = 89)	Female (*n* = 74)	*P*
Dairy	365.40 (249.13, 551.76)	392.49 (250.63, 614.02)	326.81 (248.10, 475.76)	0.162
Breads	336.09 (228.66, 474.85)	353.89 (233.22, 553.76)	304.72 (225.95, 444.95)	0.199
Savory snacks	244.82 (120.17, 385.32)	297.90 (148.45, 489.95)	180.29 (105.16, 368.49)	0.013
Poultry	240.82 (121.22, 377.84)	247.26 (121.22, 508.90)	218.78 (121.22, 363.67)	0.277
Pizza	222.71 (93.95, 416.99)	264.55 (93.95, 438.75)	198.53 (87.96, 343.23)	0.533
Cold cuts	135.35 (90.23, 223.32)	135.35 (83.77, 233.60)	135.35 (90.23, 216.56)	0.742
Pasta mixed dishes	123.58 (54.98, 248.20)	147.72 (59.70, 269.62)	87.57 (45.46, 218.38)	0.031
Sandwiches	123.00 (94.86, 234.93)	123.00 (102.86, 236.35)	123.00 (80.70, 202.29)	0.663
Meat mixed dishes	47.23 (26.77, 88.74)	67.37 (37.40, 100.38)	44.57 (18.23, 70.87)	0.006
Oils	45.53 (11.73, 94.29)	45.36 (11.01, 94.64)	47.14 (11.93, 90.80)	0.996
Soups	41.65 (7.00, 161.41)	105.49 (7.43, 182.62)	16.6 (4.18, 128.80)	0.270
Fish	29.49 (13.93, 64.86)	39.34 (16.71, 75.86)	25.07 (13.29, 47.36)	0.046
Eggs	27.99 (13.41, 56.81)	37.20 (16.79, 56.81)	23.41 (9.99, 53.59)	0.262
Beverages	21.69 (11.91, 35.19)	22.76 (12.84, 39.05)	19.85 (9.49, 30.38)	0.160

*Results are presented as P50 (P25, P75) for skewed variables. *P*-values derived through the Mann–Whitney *U* test, after controlling for the normality of the distribution*.

## Discussion

Sodium and potassium intakes are evaluated herein from food diaries and 24-h urine collections over a 7-day period, while fluctuation in sodium and potassium intake in 6-h intervals is observed. This approach provides additional information to that from single 24-h urine samples, which is considered the “gold standard” method for assessing sodium and potassium intakes. This is the first time that important insights and extended information on sodium and potassium intake are provided for free living population.

In the studied population, mean urine sodium excretion was 2,803.3 ± 1,249.0 mg/day (121.9 ± 54.3 mmol/day) and mean urine potassium excretion was 2,152.2 ± 913.3 mg/day (55.2 ± 23.4 mmol/day). These findings are in accordance with or deviate slightly from previous studies that use 24-h urine collection. In particular, in the INTERMAP study (32), conducted in the UK, urine sodium was 161 mmol/day in men and 127 mmol/day in women. In a subsample of the PURE study with 1,083 subjects from 11 countries (33), urine sodium was 4,116 ± 1,978 mg/day. In 148 Australian parents, urine sodium and potassium were 120 ± 45 and 68 ± 19 mmol/day for females; 152 ± 49 and 91 ± 40 mmol/day for males, respectively (34). Data for the Greek population are limited. An older study conducted in 50 boys aged 8–9 years in Greece reported a 24-h urinary sodium excretion of 112.0 mmol/day (35). In the recent study in Northern Greece urine sodium and potassium were 174.7 ± 72.2 and 65.1 ± 24.6 mmol/day, respectively (25).

Spot urine collections have been proposed (spot, timed, daytime, evening, overnight) as alternative to 24-h urine collection to address practical difficulties (36), associated with reduced response rate of participation or errors in adherence to the protocol (37). Partial correlations of spot urine collections are highly variable at the individual level; in our study analysis of sodium, potassium and creatinine in 6-h intervals confirmed fluctuations during the day. We found that the highest potassium concentration was measured in the afternoon, while the lowest sodium concentration was measured in the overnight 6-h interval. Metabolic studies indicate that, in healthy adults, most of the sodium consumed is excreted in the afternoon and in the evening with sodium excretion dipping to low levels from midnight to early morning (38, 39). To account for fluctuation during the day, Cohall et al. (40) proposed the use of an afternoon 12-h timed sample and not a spot afternoon sample for the estimation of 24-h sodium excretion. The Kawasaki method (26), the Tanaka method (27), and the INTERSALT method (24) propose equations to estimate the 24-h sodium excretion, using data from second urine or spot urine samples. These equations were developed in Asian populations and may not apply in Western or US populations (9); we may confirm that estimated sodium in 24-h urine, using data from second urine or spot urine samples deviate from measured values.

Food sodium intake, estimated from food diaries, was 1,983.2 ± 814.1 and food potassium was 2,264.5 ± 653.3 mg/day in our study. Previous studies recorded higher sodium intake; for example, 3,030 (2,204, 4,286) mg/day (41) in Canadian healthy adults, 4.7 ± 2.6 g/day in Chinese adults (42), 2,654 ± 540 mg/day in Iranian hypertensive adults (43), or 2,436 and 1,796 mg/day in Greek boys and girls, respectively (20). This could be explained by the fact that we did not estimate the salt added at the table employing specific methodology (44). Moreover, sodium intake may be underestimated when diary records are used; this could be due to inaccurate quantification of the amount of salt added at the table or during cooking and variation in the sodium content of processed food (9). New research developments that introduce electronic recording of dietary intake attempt to improve dietary assessment methods (45).

Food sodium intake measurements suggest that the adherence of this study population to WHO recommendations concerning sodium intake (<2,000 mg/day) was 58%. However, this percentage drops to 36% when sodium intake was calculated to 3,048.02 ± 1,411.24 mg/day multiplying 24-h urine measurements with 1.05 according to Vasara et al. (25). This percentage is still higher than that in Northern Greece, in which only 5% of the participants met WHO recommendations for sodium intake Vasara et al. (25) and in the USA (46). Moreover, only 7.4% of subjects met WHO recommendations for potassium intake. Various approaches have been proposed in order to reduce food sodium intake with simultaneous increase of food potassium intake, including the replacement of sodium chloride by potassium chloride in bread, processed fruit and vegetables, snacks and processed meat (47). Strategies for salt reduction and potassium integration in the diet should continue. Japan, UK, Finland, Portugal, Greece, USA, and Canada implemented strategies in order to raise consumers’ awareness and limit sodium intake. They suggested labeling their products with sodium concentration, so that consumers can easily distinguish and choose products low in sodium. In addition, with the cooperation of food industry and food service providers they also reformulate their products and meals in order to reduce the sodium content. Campaigns have been organized in order fresh products to be preferred to processed ones, home cooked dishes to restaurant prepared meals and natural herbs to salt (6, 48, 49).

In our study, food sources that contributed most to food sodium intake were dairy products 24%, breads 22% and savory snacks 17%. It is proposed that approximately 75% of sodium intake comes from processed or restaurant foods, 10–12% is endogenous in foods and the remaining 10–15% comes from the discretionary use of salt added at the table or while cooking (13, 14). In UK cereals and cereal products including also bread, biscuits and crackers contributed approximately 38% of total sodium intake, while meat and meat products about 21% (15). In the USA breads and rolls, cold cuts, pizza, poultry, soups and sandwiches contributed approximately 30% of daily sodium consumption (16). In a sample of 655 Chinese women, the food groups of soups (22%), rice, and noodles (14%) (50) were contributed in major to non-discretionary salt. In general, in Asian countries most of the sodium intake comes from salt added during cooking and from sauces and seasonings (15). It appears that there is a noticeable variation among different populations and this may be attributed to different dietary and cultural habits.

Sodium and potassium intakes were positively correlated with energy intake. This finding reveals the dietary patterns related to sodium intake and is in line with previous observations (51). Food sodium intake correlated with urine sodium in the evening 6-h interval urine collection.

Notable differences between males and females were observed. A higher food sodium and potassium intake was observed in males compared with females. This finding is in accordance with a study conducted in US population (52) and in Korean population (53). Moreover, a higher contribution of meat mixed dishes in sodium intake was observed in men compared with women. Also, men consumed higher amounts of savory snacks and pasta mixed dishes compared with women, resulting in a higher contribution of these food groups to sodium intake. Similar observations were recorded in Italy where the consumption of meat was significantly higher in men (917 g per week) compared with women (679 g per week) (54). However, it should be mentioned that no differences were observed in sodium intake density between males and females (1,186 and 1,273 mg/1,000 kcal, respectively, *p* > 0.005). This finding is in accordance with previous studies (12, 55) that no differences were observed between sexes.

We observed weak to moderate correlations between food sodium and potassium intake and sodium and potassium in urine in 24-h samples but not in all 6-h intervals measured. This does not fully agree with (56) who suggested that urine samples collected post prandially (2–4 h after the consumption) reflect dietary habits. This may be due to a variety of factors, such as the design of our study, where urine specimens were separated in 6-h intervals, misestimation of sodium and potassium intake from food diaries, non-estimation of salt added at table, or interactions between nutrients that affect excretion physiology. More studies with shorter time periods and more stringent protocol which will overcome the underestimation of intake and deal with the overlapping of urinary time periods, should explore the relation between intake and excretion of sodium and potassium in healthy adults. There are some limitations in the present study that should be noted. The 24-h urine collection and food recording for seven consecutive days has a high burden for the subjects; this may affect their dietary behavior during the experimental period (57). The estimation of sodium intake using food diaries included sodium intake in fresh, frozen, processed food, and in meals as calculated from Food Composition databases and it not include table salt. It must be also mentioned that, because of our recruitment methodology, the sample was not representative to the Greek population therefore data may be interpreted with caution. This study is not representing the whole population; however, the correlations between different measurements highlight the availability of different indices to obtain information about sodium intake.

## Conclusion

In conclusion, data for food sodium intake from 7-day diaries indicate that sodium intake is higher than WHO recommendations for 40% of the studied population. It should be noted that this percentage was calculated without the inclusion of table salt. This high-sodium consumption is also confirmed in data on sodium excretion from 24-h urine collection for seven consecutive days in Greece, averaging 2,803.3 ± 1,249.0 mg/day. Additionally, potassium intake still remains lower than recommendations. The main sources of sodium in our study were dairy products, breads, and savory snacks. Further research on a representative sample of the Greek healthy adult population should be performed in other to understand its dietary sodium and potassium pattern. Strategies should encourage the Greek population to moderate sodium intake and increase food potassium intake, thus adopting a healthier dietary and lifestyle pattern.

## Ethics Statement

This study was carried out in accordance with the recommendations of the Research Ethics Committee of Agricultural University of Athens, Greece (197/27-02-2012). This study was approved by the Research Ethics Committee of Agricultural University of Athens, Greece. All subjects gave written informed consent in accordance with the Declaration of Helsinki. Details that might disclose the identity of the subjects under study were omitted.

## Author Contributions

AA, AK, and MK formulated the research questions. AA, AK, OM, AP, and MK designed the study. AA, AK, OM, and AP carried out the study. AA and KA analyzed the data. AA, AK, and MK wrote the paper. All authors were substantial contributors to the conception or design of the work; or the acquisition, analysis, or interpretation of data for the work. They all drafted the work revised it critically for important intellectual content. All of them gave their final approval of the version to be published. Finally, they all agreed to be accountable for all aspects of the work in ensuring that questions related to the accuracy or integrity of any part of the work are appropriately investigated and resolved.

## Conflict of Interest Statement

The authors declare that the research was conducted in the absence of any commercial or financial relationships that could be construed as a potential conflict of interest.
